# The association between county-level premature cardiovascular mortality related to cardio-kidney-metabolic disease and the social determinants of health in the US.

**DOI:** 10.1038/s41598-024-73974-9

**Published:** 2024-10-23

**Authors:** Antoinette Cotton, Pedro R V O Salerno, Salil V Deo, Salim Virani, Khurram Nasir, Ian Neeland, Sanjay Rajagopalan, Naveed Sattar, Sadeer Al-Kindi, Yakov E Elgudin

**Affiliations:** 1 Case Western Reserve University School of Medicine, Cleveland, USA; 2 Harrington Heart and Vascular Institute, University Hospitals, Cleveland, USA; 3 Louis Stokes Cleveland VA Medical Center, 10701 East Boulevard, Cleveland, OH 44106 USA; 4 School of Health and Wellbeing, University of Glasgow, Glasgow, UK; 5 The Aga Khan University, Karachi, Pakistan; 6 Baylor College of Medicine, Houston, USA; 7 DeBakey Heart and Vascular Center, Houston Methodist Hospital, Houston, USA; 8 School of Cardiovascular and Metabolic Health, University of Glasgow, Glasgow, UK; 9 Cardiovascular Prevention & Wellness Houston Methodist DeBakey Heart & Vascular Center, 6550 Fannin Street, Suite 1801, Houston, TX 77030 USA

**Keywords:** Cardiovascular disease, Kidney disease, Renal failure, Diabetes, Obesity, Cardiometabolic health, Cardiology, Health care

## Abstract

Cardio-kidney-metabolic (CKM) syndrome is defined by the American Heart Association as the intersection between metabolic, renal and cardiovascular disease. Understanding the contemporary estimates of CKM related mortality and recent trends in the US is essential for developing targeted public interventions. We collected state-level and county-level CKM-associated age-adjusted premature cardiovascular mortality (aaCVM) (2010–2019) rates from the CDC Wide-ranging Online Data for Epidemiologic Research (WONDER). We linked the county-level aaCVM with a multi-component social deprivation metric: the Social Deprivation Index (SDI: range 0–100) and grouped them as follows: I: 0–25, II: 26–50, III: 51–75, and IV: 76–100. We conducted pair-wise comparison of aaCVM between SDI groups with the multiplicity adjusted Wilcoxon test; we compared aaCVM in men versus women, metropolitan versus nonmetropolitan counties, and non-hispanic white versus non-hispanic black residents. In 3101 analyzed counties in the US, the median CKM associated aaCVM was 61 [interquartile range (IQR): 45, 82]/100 000. Mississippi (99/100 000) and Minnesota (33/100 000) had the highest and lowest values respectively. CKM associated aaMR increased across SDI groups [I – 45 (IQR: 36, 55)/100 000, II- 61 (IQR: 49, 77)/100 000, III- 77 (IQR: 61, 94)/100 000, IV- 89 (IQR: 70, 110)/100 000; all pair-wise p-values < 0.001]. Men had higher rates [85 (64, 91)/100 000] than women [41 (28, 58)/100 000](p-value < 0.001), metropolitan counties [54 (40, 72)/100 000] had lower rates than non-metropolitan counties [66 (49, 90)/100 000](p-value < 0.001), and non-Hispanic Black [110 (86, 137)/100 000] had higher aaMR than non-Hispanic White residents [59 (44, 78)/100 000](p-value < 0.001). In the US, CKM mortality remains high and disproportionately occurs in more socially deprived counties and non-metropolitan counties. Our inability to reduce CKM mortality rates over the study period highlights the need for targeted policy interventions to curb the ongoing high burden.

**Supplementary Information:**

The online version contains supplementary material available at 10.1038/s41598-024-73974-9.

## Introduction

The American Heart Association (AHA) recently introduced a combined phenotype labeled the cardiovascular-kidney-metabolic (CKM) syndrome^1^. Most factors responsible for developing incident cardio-renal disease are reversible. Therefore, appropriately preventing or treating these risk factors can reduce the rate of downstream adverse cardiovascular events. A recent study reported that the premature cardiovascular mortality (defined as death prior to 65 years) rate has reduced till 2011 but remained stagnant since then^2^. However, in the recent decade, the rates of obesity and diabetes have reached epidemic proportions in the US^3^. Therefore, the current burden of the CKM syndrome related mortality in the US is unknown. This is even more important given the recent introduction of novel cardio- and kidney-protective agents like GLP1RA (glucagon-like protein 1 receptor agonists) and SGLT2i (sodium glucose L-type transport protein 2 inhibitors). Recent randomized trials have reported that these drugs reduce the rates of myocardial infarction, stroke, and heart failure among patients with diabetes. GLP1RA drugs have also reported excellent weight loss benefits and have reduced adverse cardiovascular event rates in obese people without diabetes. Given these cardiovascular benefits, based on recommendations from the American Diabetes Association, either or both agents would now be considered first-line drugs for a large proportion of CKM patients^4^. However, apart from the traditional clinical cardiovascular risk factors like hypertension, diabetes, obesity and dyslipidemia, recent research has also firmly demonstrated that the social determinants of health (SDoH) play an increasingly important role in determining incident premature cardiovascular mortality in the US^5,6^. While these studies reported higher cardiovascular mortality rates among socially vulnerable counties in the US, this question has not been evaluated in people with underlying CKM. The first step towards population-level health improvement is clearly understanding the problem burden. Such information will inform us to direct healthcare resources towards more vulnerable counties in the US. We, therefore, sought to characterize the rates of CKM associated premature mortality (ages 15–64) in the US, evaluating it at both the state- and county-level. We further linked the county-level data with a multi-component publicly available social deprivation score to examine the demographic and spatial relationships between to the social determinants of health and CKM associated premature aaCVM burden in the US.

## Methods

### County-level aaCVM

We used the multiple causes of death query portal to obtain the state-level and county-level age-adjusted rates reported between 2010 and end 2019 from the Center for Disease Control (CDC) WONDER (Wide-ranging ONline Data for Epidemiologic Research). This data portal, which collates information from state death certificate records, was developed by the CDC to inform public health initiatives regarding epidemiologic trends of disease related mortality in the US. In CDC WONDER’s multiple causes of death field, we chose the international classification of disease 10th version codes (ICD 10th ) corresponding to the clinical conditions that comprise the AHA’s description of the components of CKM (Table S1). We selected the ICD 10th version codes for cardiovascular disease in the underlying cause of death field. To obtain only premature aaCVM, we limited the age query. We defined premature death as death certificates of adults less than 64 years of age^2,7,8^. We also collected these rates for the following sub-groups: sex (men and women), county location (metropolitan and non-metropolitan), race/ethnicity [non-Hispanic White, non-Hispanic Black, Hispanic, and other race/ethnicity (pooled data from American Indian, Alaskan Natives, and Asians)]. We limited our query years till end 2019 to avoid the undue influence of the Covid19 pandemic on the reported aaCVM rates. The CDC portal automatically suppressed results when the crude death counts in the county were very small, and we limited our analysis to counties that reported data for each studied group.

### Social Deprivation Index

 The Social Deprivation Index (SDI) is a single score that is representative of the social determinants of health that prevail in that population^9^. The Robert Graham Center (https://www.graham-center.org/home.html), a non-profit organization, first designed this score to inform population level healthcare delivery. The SDI is a composite metric of socioeconomic status and contains 7 domains, specifically: poverty (percentage of residents below the poverty level), education (percent population with less than a high-school education), employment (percentage of unemployed adults), housing (percentage of households living in rental units), overcrowding (percentage of households living in crowded housing units), single parent families (percentage of single parent families with young dependents < 18 years old), and vehicular access (percentage of households without a vehicle). The SDI ranges between 0 and 100, with higher values depicting increased social deprivation. We used SDI values from only one year: 2012. We further grouped counties based on their SDI as follows: Group I: SDI 0–25, Group II: SDI 26–50, Group III: SDI 51–75, Group IV: SDI 76–100. To ensure that the simplification of using only one year was appropriate, we conducted a sensitivity analysis of the mortality rate using SDI assignments from 2016 to 2018. This analysis showed minimal change in mortality rates across SDI quartiles when the SDI reference year was changed (Table S2).

### Statistical analysis

We obtained the CKM associated aaCVM rate (per 100,000 residents) for each state and county in the US (2010–2019). As the aaCVM rates were not normally distributed, we reported all data using the median and the inter-quartile range. We then obtained the CKM associated aaCVM for each SDI group. We performed group-wise comparison of the aaCVM using the pairwise Wilcoxon test and adjusted for multiple group comparison hypotheses with the Bonferroni method. We further performed the above analyses in the following subgroups: men and women residents, metropolitan and non-metropolitan counties, race, and ethnicity (non-Hispanic White residents, non-Hispanic Black residents, Hispanic residents, and residents of other races). Finally, as a sensitivity analysis to investigate the adjusted association between SDI and aaCVM rates, we collected the county-level prevalence of obesity, diabetes, hypertension, and hypercholesterolemia from CDC PLACES (2023), a publicly available data set that provides estimations of chronic diseases for the entire US^10^. We then fit a generalized linear regression model with a Poisson link with the aaCVM rates as the dependent variable and the county SDI as primary exposure. We obtained the coefficients for each SDI quartile (with the 1st quartile as the reference) and calculated the robust standard errors at the 95% confidence level.

We used R 4.2.2 (The R Foundation for Statistical Computing) for all statistical analyses. To create the US maps we imported cartographic boundary shape files from the US Census (https://www.census.gov/geographies/mapping-files/time-series/geo/tiger-line-file.html) and used the ‘sf’ package. We conducted and reported the study according to STROBE guidelines. We did not need to obtain institutional review board approval for this study as it used publicly available and aggregated data.

## Results

**Fig. 1:**
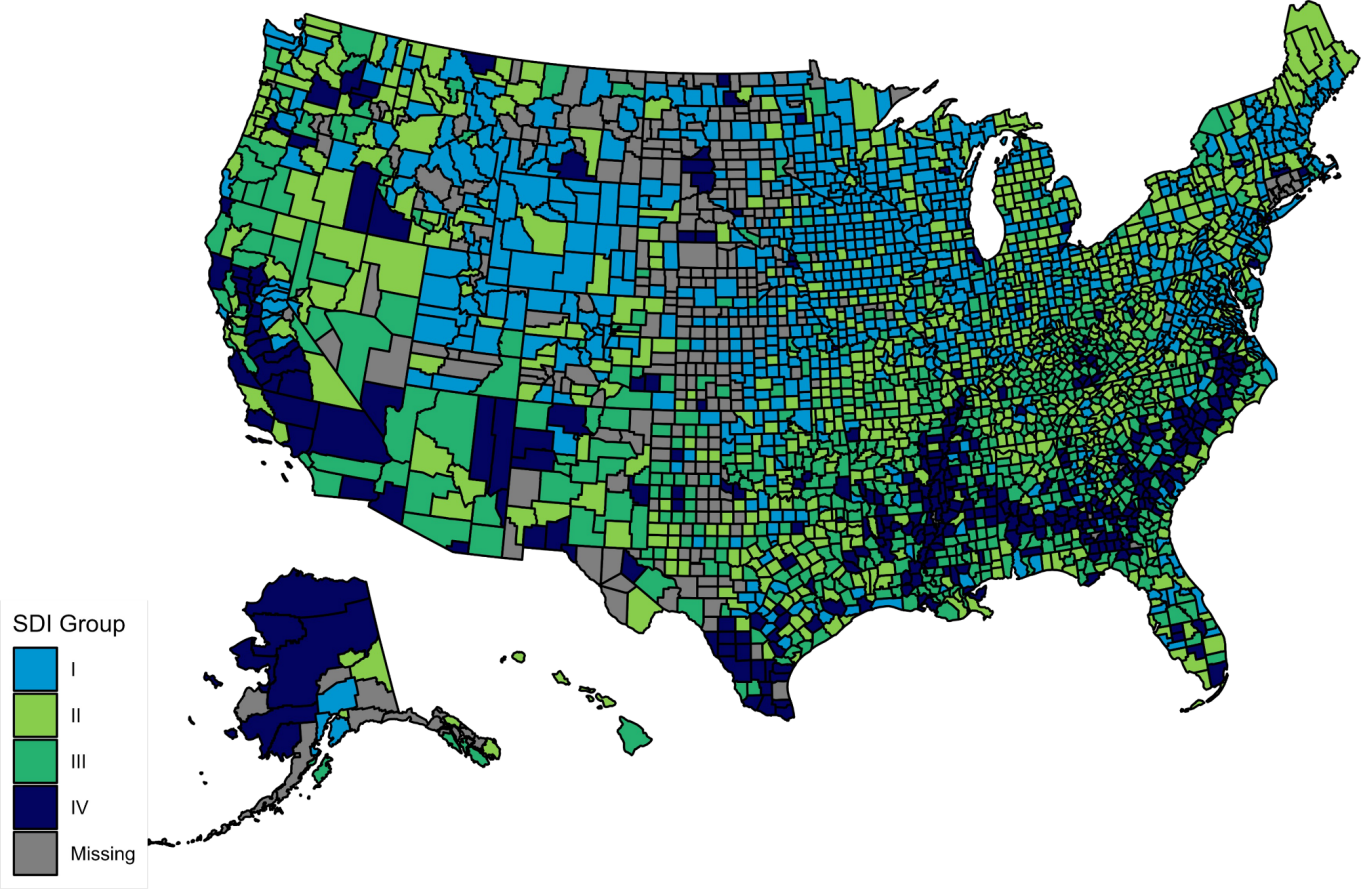
Social deprivation index across US counties. This map presents the SDI group that each US county in our study belongs to. The counties that are excluded in our study have been grayed out. Abbreviation: SDI – social deprivation index

**Fig. 2:**
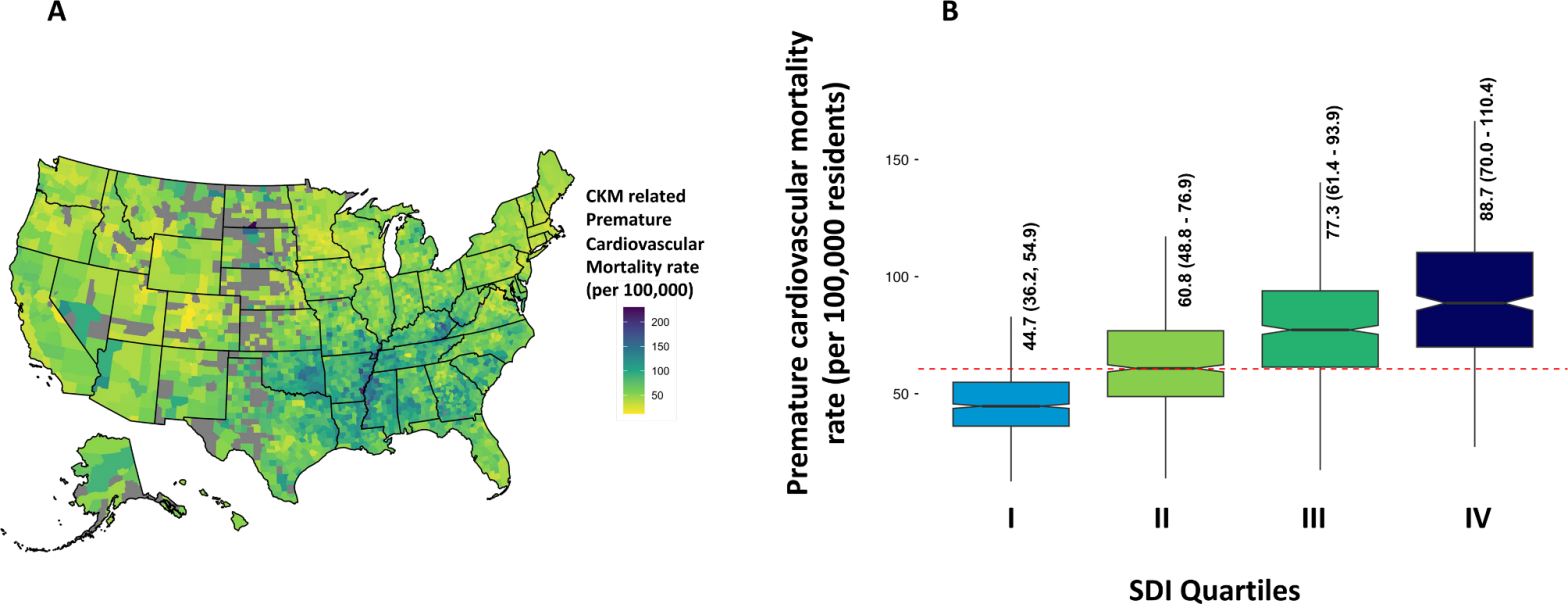
Title: County-level CKM related premature age adjusted cardiovascular mortality in the US. (**A**) This map presents the county-level median CKM related premature aaCVM rate reported in each county that we studied. (**B**) This grouped boxplot presents the distribution of the CKM related premature aaCVM rates for each SDI group. The middle of each boxplot notch corresponds to the median value, the lower and upper ends of the box correspond to the 1st and 3rd quartile, and the lines extend to include 1½ times the interquartile range at each end of the distribution.

**Fig. 3:**
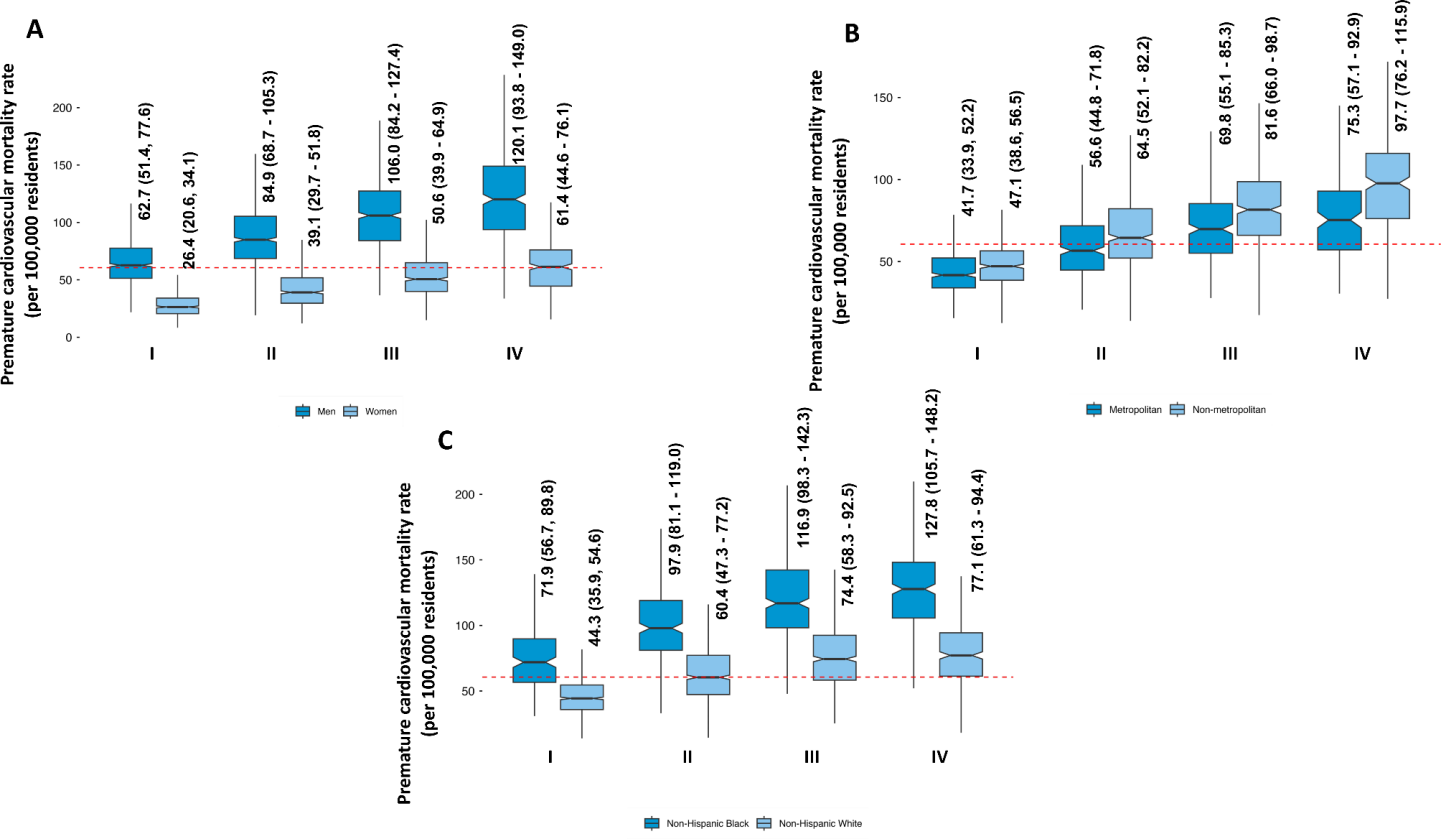
County-level CKM related premature age adjusted cardiovascular mortality in the US according to the SDI according to sex, location, and race. These grouped boxplots report CKM related aaCVM rates according to the SDI groups for (**A**) men and women, (**B**) metropolitan and non-metropolitan counties, and (**C**) non-Hispanic White and non-Hispanic Black residents. The middle of each boxplot notch corresponds to the median value, the lower and upper ends of the box correspond to the 1st and 3rd quartile, and the lines extend to include 1½ times the interquartile range at each end of the distribution. Abbreviations: aaCVM – age adjusted cardiovascular mortality, CKM – cardio-kidney-metabolic syndrome, SDI – social deprivation index

**Fig. 4:**
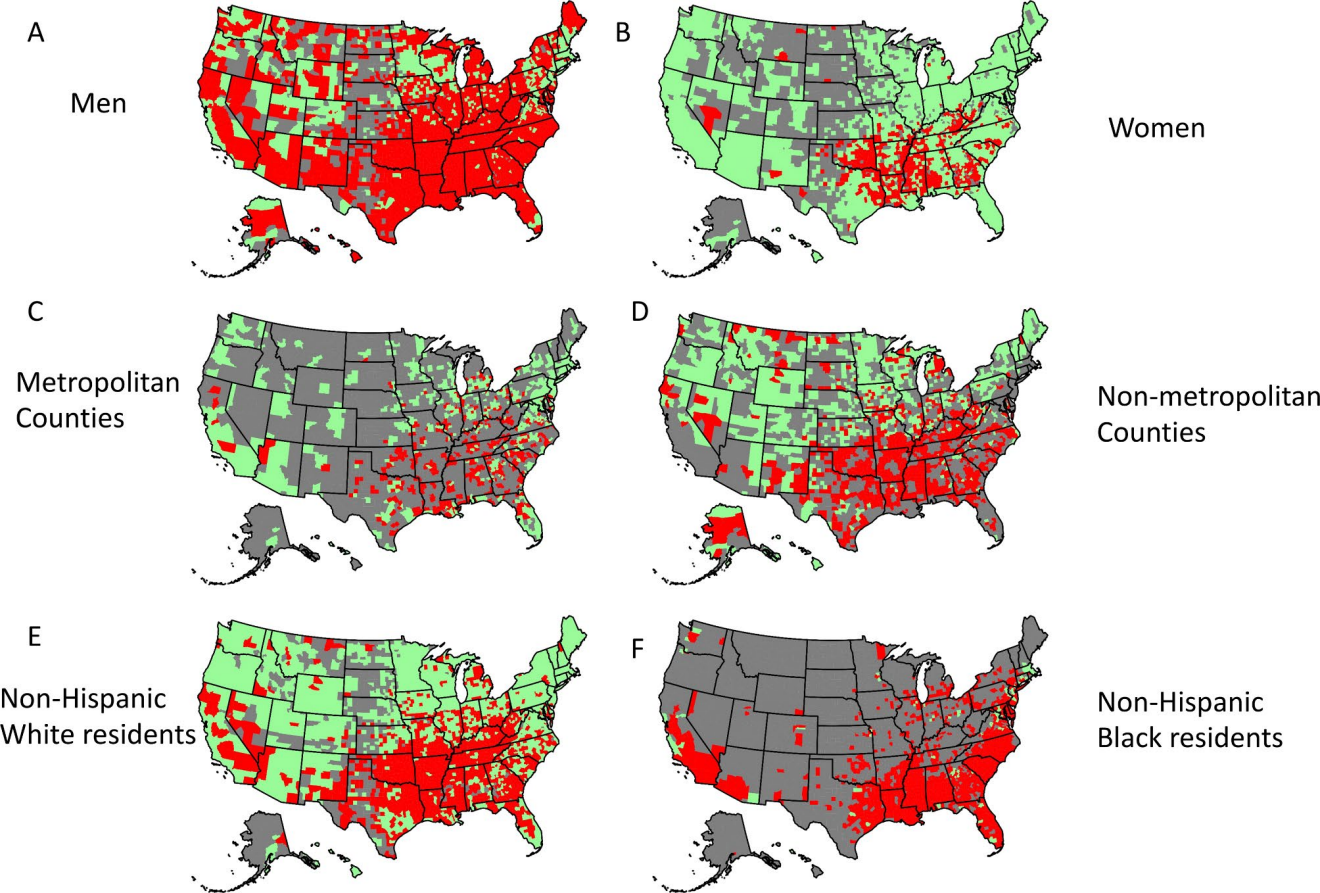
US counties that reported median CKM related premature age adjusted cardiovascular mortality rates above the national rate. In this maps panel, counties that reported median CKM related aaCVM rates above the national rate are colored red while the others are colored green. Counties not included in the analysis are grayed out in each map.

*Age adjusted mortality for CKM*: From a total of 3243 county and county equivalent regions in the US, we analyzed data from 2857 US counties (88% of the total) over the ten-year study duration (2010–2019). Among studied counties, 934 (33%), 824 (29%), 696 (24%) and 403 (14%) belonged to SDI groups I, II, III and IV respectively (Fig. 1). The median CKM related aaCVM in the US was 60.7 (Interquartile Range [IQR]: 45.5, 82.3)/100 000 residents. Mississippi [98.9 (IQR: 97.6, 100.3)] followed by Oklahoma [91.4 (IQR: 90.3, 92.5)]/ 100 000 residents had the highest CKM related aaCVM rates while Minnesota [32.5 (IQR: 32.0, 33.0)]/ 100 000 residents had the lowest rate (Table 1, Figure S1). CKM related aaCVM rates were high in many counties in the South (Fig. 2A). The CKM related aaCVM rates increased incrementally across the SDI groups: Group I – 44.7 (IQR: 36.2, 54.9)/100 000 residents, Group II- 60.8 (IQR: 48.8, 76.9)/100 000 residents, Group III- 77.3 (IQR: 61.4, 93.9)/100 000 residents, and Group IV- 88.7 (IQR: 70.0, 110.4)/100 000 residents (Fig. 2B). Each subsequent SDI group had significantly higher aaCVM rates than the prior group [p-values: II vs. I - <0.001, III vs. II: < 0.001, IV vs. III: < 0.001] (Table 2; Fig. 3A). The CKM related aaCVM was significantly higher for men [84.9 (IQR: 64.0, 91.2)]/100 000 residents than women [40.7 (IQR: 28.1, 57.6)]/100 000 residents (*p* < 0.001). Among men, Mississippi [133.9 (IQR: 131.7, 136.1)/100 000 residents] followed by Arkansas [120.6 (IQR: 118.5, 122.6)/100 000 residents] had the highest aaCVM rates while Utah [45.4 (IQR: 44.0, 46.2)/100 000 residents] had the lowest rate (Figure S2 – A). Among the 2733 counties that reported results for men, 2149 (78%) counties had median CKM related aaCVM rates that were higher than the national rate (Fig. 4A). In fact, among men, very few counties in each US state had CKM related aaCVM rates lower than the national rate. In women, Mississippi [66.6 (IQR: 65.1, 68.1)/100 000 residents] followed by Oklahoma [63.5 (IQR: 62.2, 64.8)/100 000 residents] had the highest CKM related aaCVM rates, while Massachusetts [17.7 (IQR: 17.2, 18.2)/100 000 residents] reported the lowest rate in the nation (Figure S2 – B). Among the 2280 counties that reported data for women, 488 (17%) had median CKM related aaCVM rates above the national rate (Fig. 4B). Furthermore, these counties were largely located as a band across the southern US. In both men and women, the aaCVM rates increased across the SDI and the pairwise comparison between all groups in both men and women were statistically significant (Table 2). Additionally, for each SDI group, men had much higher aaCVM rates than women (Fig. 3A).

**Table 1:** States with the highest and lowest premature age-adjusted cardiovascular mortality rates associated with cardio-kidney-metabolic syndrome in the US (2010–2019).

	Location	Median all-cause mortality related to CKM (Interquartile range) per 100 000 residents
Overall cohort		
Highest:	Mississippi	98.9 (97.6-100.3)
	Oklahoma	91.4 (90.3–92.5)
	Arkansas	89.5 (88.2–90.8)
Lowest	Utah	32.8 (32.0-33.6)
	Massachusetts	32.7 (32.2–33.2)
	Minnesota	32.5 (32.0–33.0)
Men		
Highest	Mississippi	133.9 (131.7-136.1)
	Arkansas	120.6 (118.5-122.6)
	Oklahoma	120.3 (118.5-122.1)
Lowest	Massachusetts	48.7 (47.9–49.6)
	Minnesota	46.7 (45.8–47.7)
	Utah	45.4 (44.0-46.8)
Women		
Highest	Mississippi	66.6 (65.1–68.1)
	Oklahoma	63.5 (62.2–64.8)
	District of Columbia	60.6 (57.4–63.8)
Lowest	Minnesota	18.4 (17.8–19.0)
	New Hampshire	18.3 (17.1–19.4)
	Massachusetts	17.7 (17.2–18.2)
Metropolitan counties only		
Highest	Mississippi	89.4 (87.5–91.2)
	Arkansas	84.6 (83.1–86.2)
	Oklahoma	84.5 (83.2–85.9)
Lowest	Massachusetts	32.7 (32.2–33.2)
	Utah	32.3 (31.4–33.1)
	Minnesota	30.9 (30.3–31.5)
Non-metropolitan counties only		
Highest	Mississippi	107.2 (105.3-109.1)
	Oklahoma	104.4 (102.3-106.4)
	Louisiana	100.4 (97.7-103.1)
Lowest	Colorado	36.1 (34.5–37.6)
	Massachusetts	33.9 (29.9–37.9)
	Connecticut	33.5 (30.6–36.4)
Non-Hispanic White Residents		
Highest	Oklahoma	88.8 (87.5–90.0)
	Arkansas	85.2 (83.8–86.6)
	Mississippi	80.3 (78.7–81.8)
Lowest	Utah	32.7 (31.8–33.6)
	Minnesota	30.3 (29.7–30.8)
	District of Columbia	18.1 (15.9–20.3)
Non-Hispanic Black		
Highest	Oklahoma	147.7 (142.4–153.0)
	Michigan	138.3 (136.0-140.6)
	Arkansas	136.5 (132.4-140.6)
Lowest	Idaho	47.1 (32.2–66.5)
	North Dakota	46.5 (32.0-65.4)
	Maine	33.9 (23.3–47.6)

We collected data from the CDC Wide-ranging Online Data for Epidemiologic Research (WONDER) portal and analyzed the rates of CKM related premature aaCVM (2010–2019). In this table, we present the three US states with the highest and lowest CKM related premature aaCVM rates (per 100 000 residents) in the US.

Abbreviations: CDC – Center for Diseases Control, CKM – cardio-kidney-metabolic syndrome, aaCVM – age adjusted cardiovascular mortality

**Table 2:** County-level age-adjusted premature cardiovascular mortality rates (per 100,000 residents) associated with cardio-kidney-metabolic syndrome according to the social deprivation index in the US (2010–2019).

Rates for the whole cohort	Social deprivation index Quartile		Q2 vs. Q1 *p*-value	Q3	Q3 vs. Q2 *p*-value	Q4	Q4vs Q3 *p*-value
	Q1	Q2					
All US counties (*n* = 3,101)							
60.7 (45.5, 82.3)	44.7 (36.2–54.9)	60.8 (48.8–76.9)	< 0.01	77.3 (61.4–93.9)	< 0.01	88.7 (70.0–110.4)	< 0.01
All US counties; only men							
84.9 (64.0, 91.2)	62.7 (51.4, 77.6)	84.9 (68.7–105.3)	< 0.01	106.0 (84.2–127.4)	< 0.01	120.1 (93.8–149.0)	< 0.01
All US counties; only women							
40.7 (28.1, 57.6)	26.4 (20.6, 34.1)	39.1 (29.7–51.8)	< 0.01	50.6 (39.9–64.9)	< 0.01	61.4 (44.6–76.1)	< 0.01
Only metropolitan counties							
54.3 (40.2, 72.1)	41.7 (33.9, 52.2)	56.6 (44.8–71.8)	< 0.01	69.8 (55.1–85.3)	< 0.01	75.3 (57.1–92.9)	0.02
Only non-metropolitan counties							
66.3 (49.0, 89.5)	47.1 (38.6, 56.5)	64.5 (52.1–82.2)	< 0.01	81.6 (66.0–98.7)	< 0.01	97.7 (76.2–115.9)	< 0.01
All US counties; non-Hispanic white residents							
58.8 (44.2, 78.0)	44.3 (35.9, 54.6)	60.4 (47.3–77.2)	< 0.01	74.4 (58.3–92.5)	< 0.01	77.1 (61.3–94.4)	0.09
All US counties; non-Hispanic black residents							
110.1 (85.6, 136.5)	71.9 (56.7, 89.8)	97.9 (81.1–119.0)	< 0.01	116.9 (98.3–142.3)	< 0.01	127.8 (105.7–148.2)	< 0.01

We collected data from the CDC Wide-ranging Online Data for Epidemiologic Research (WONDER) portal and analyzed the rates of CKM related premature aaCVM (2010–2019). We further grouped counties based on their SDI into four groups and fit pair-wise comparisons between these groups to compare the CKM related premature aaCVM rates. In this table, we present the CKM related aaCVM rates for each SDI group and three US states with the highest and lowest CKM related age adjusted cardiovascular mortality rates (per 100 000 residents) in the US.

aaCVM – age adjusted cardiovascular mortality, CDC – Center for Diseases Control, CKM – cardio-kidney-metabolic syndrome

The aaCVM rates in metropolitan counties [1150 counties, median rate 54.3 (IQR: 40.2, 72.1) /100 000 residents] were lower than in non-metropolitan counties [1707 counties; median rate 66.3 (IQR: 49.0, 89.5) /100 000 residents] (p-value < 0.001). Among metropolitan counties, Mississippi [89.4 (IQR: 87.5, 91.2) /100 000 residents] followed by Arkansas [84.6 (IQR: 83.1, 86.2) /100 000 residents] had the highest aaCVM rates, while Minnesota [30.9 (IQR: 30.3, 31.5) /100 000 residents] reported the lowest rate in the nation (Figure S3 – A). In the 1150 metropolitan counties that reported data, 459 (39%) had median CKM related aaCVM rates above the national rate (Figs. 3B and 4C). Most of these metropolitan counties were in the South. Among non-metropolitan counties, Mississippi [107.2 (IQR: 105.3, 109.1) /100 000 residents] followed by Oklahoma [104.4 (IQR: 102.3, 106.4) /100 000 residents] had the highest aaCVM rates, while Connecticut [33.5 (IQR: 30.6, 36.4) /100 000 residents] reported the lowest rate in the nation (Figure S3 – B). Among the 1707 non-metropolitan counties, 976 (57%) reported median CKM related aaCVM rates above the national rate (Figs. 3B and 4D). In metropolitan counties, aaCVM rates increased significantly across SDI group I, II and III; however, aaCVM rates between SDI group III and IV were slightly more similar (p-value 0.02) (Fig. 3B; Table 2). In non-metropolitan counties on the other hand, aaCVM rates increased significantly across the SDI groups (Fig. 3B; Table 2).

Overall, in the US, non-Hispanic Black residents [1088 counties, median aaCVM rate 110.1 (IQR: 85.6, 136.5) /100 000 residents] had almost double the rates of aaCVM as non-Hispanic White residents [2754 counties, median aaCVM rate 58.8 (IQR: 44.2, 78.0) /100 000 residents]. Hispanic (median rate 34.2/100 000 residents) and residents of other races (Asian, Alaskan Natives, Pacific Islanders, American Indian) (median rate 35.4/100 000 residents) had low aaCVM rates. However, given the low number of residents belonging to these racial/ethnic minorities in many counties, we only obtained data regarding Hispanic and residents of other races from 437 (13% of all US counties and county equivalent regions) and 336 (10% of all US counties and county equivalent regions) counties respectively. For non-Hispanic White residents, Oklahoma [88.8 (IQR: 87.5, 90) /100 000 residents] followed by Arkansas [85.2 (IQR: 83.8, 86.6) /100 000 residents] had the highest aaCVM rates, while the District of Columbia [18.1 (IQR: 15.9, 20.3) /100 000 residents] reported the lowest rate in the nation. In non-Hispanic Black residents, Oklahoma [147.7 (IQR: 142.2, 153.0) /100 000 residents] followed by Michigan [138.8 (IQR: 136.0, 140.6) /100 000 residents] had the highest aaCVM rates, while Maine [33.9 (IQR: 23.3, 47.6) /100 000 residents] reported the lowest rate in the nation. In the 2754 counties that reported data for non-Hispanic White residents, 1295 (45%) had a median CKM related aaCVM rate above the national rate (Figs. 3C and 4E). In the 1088 counties that reported data for non-Hispanic Black residents, 1006 (92%) reported a median CKM related aaCVM rate above the national rate (Figs. 3C and 4F). In non-Hispanic black residents, aaCVM rates increased significantly across all SDI groups (all pairwise comparison p-values < 0.01), while in non-Hispanic white residents, aaCVM rates in group II > group I, and group III > group II, but rates in groups III and IV were slightly more similar (p-value 0.09) (Fig. 3C).

On sensitivity analysis, after adjusting for obesity, diabetes, hypertension, and hypercholesterolemia, we found that there remained a significant association between SDI group (coefficients for SDI quartile II: 0.169, *p* < 0.001, quartile III: 0.228, *p* < 0.001, and quartile IV: 0.200, *p* < 0.001) and aaCVM.

## Discussion

### Salient findings

We analyzed state- and county-level aaCVM rates with underlying CKM syndrome from the CDC WONDER registry (2010–2019) and studied the association of aaCVM with the level of social deprivation, as measured by the social deprivation index. We observed substantial variation in the aaCVM rates in US states. Men had higher rates than women, metropolitan had higher rates than non-metropolitan counties, and non-Hispanic Black had higher rates than non-Hispanic White residents. We also observed significant incremental increase in the aaCVM rates across the SDI groups.

### Implications

 Since the early part of the 20th century, heart disease has always been an important cause of death in the US^11^. A recent study reported that age-adjusted premature (35–64 years) heart disease related mortality reduced in the US from 206/100,000 in 1968 to 62/100,000 in 2017^2^. However, since 2011 premature heart disease related mortality rates have been relatively constant^2^. Our observations also mirror those by others that reported higher heart disease related premature mortality rates among men, Black residents, and those living in rural areas. In this study, we did not report separate rates for the different conditions that comprise cardiovascular mortality. However, a study reported that while mortality rates due to coronary artery disease have declined in the US between 1980 and 2018, those related to hypertensive heart disease, cardiomyopathy, heart failure, and peripheral arterial disease are on the rise^12^. A recent modeling study projected that the US may experience a 6% relative decline in the premature mortality rate related to heart disease between 2017 and 2030^7^. Unfortunately, the projected loss of 1.6 million lives due to premature cardiovascular mortality over this time frame is still a very large number. Moreover, authors in this modeling study also report that while rates may decline among White and Black US residents, women, Hispanic, American Indians and Pacific Islanders are likely to experience a rise their premature cardiovascular mortality rates. It is indeed unfortunate that simple primary preventive measures like regular physical activity, decreasing tobacco use, and eating a well-balanced heart healthy diet are still poorly adopted in many neighborhoods. As has also been reported earlier, rural areas, racial/ethnic minorities and deprived neighborhoods are those population subgroups that continue to have despairingly low usage of these preventive strategies^13,14^. Our findings help to quantify the long-term impact of these tendencies.

However, apart from individual factors, neighborhood level parameters like poor air quality, lack of green space, and increased noise pollution are also evident in socially deprived areas in the US^15–17^. These community-level disparities are also very apparent in the adoption of secondary preventive measures like intensive blood pressure control, statin therapy for dyslipidemia, and the use of newer agents for the treatment of diabetes and heart failure.^18–21^ Tackling these inequities among the young population residing in these neighborhoods will reduce the rates of downstream cardiovascular disease and premature cardiovascular mortality. As prior research has also clearly demonstrated, public health initiatives and community driven measures can effectively promote healthy lifestyle practices^22^. The US Department of Health and Human Services therefore has an initiative ‘Healthy People 2030’ that is a data driven community-based health initiative to promote health and well-being in the next decade^23^. Our study, therefore, provides readers with the state- and county-level rates of CKM related premature aaCVM in the US. Understanding this data and the differences in distribution of rates across geography can help to inform such initiatives like the ‘Healthy People 2030’. Even for the nation as a whole, these results have major implications as the US population is aging and the active work force is shrinking. Therefore, increased premature mortality can result in increased economic burden and loss of national productivity.

### Limitations

Readers should understand our results on the background of the following limitations. It is important to note that given the retrospective and observational nature of the study, it cannot establish temporal or causal relationships among the variables and outcomes investigated. The CDC WONDER portal omits results when age adjusted rates are < 10/100 000 population. From our maps, it is clear that some sparsely populated rural counties in the Midwest were excluded from our study. This fact may limit the generalizability of our study for these small, rural counties.

The CDC WONDER reports results using information from death certificates, and the accuracy of ICD coding for conditions that comprise the CKM syndrome may vary across counties and US regions. However, ICD coding errors, if any, would in fact underestimate the true rates of premature aaCVM associated with CKM in the US. Also, we would not expect coding errors to disproportionately affect any specific SDI group. Hence, even accounting for this margin of error, the reported relationship observed between groups would remain the same. Further, given the coding changes that accompanied the COVID-19 pandemic, we chose to exclude the year 2020 from our analysis, which limited our ability to fully see trends and changes in mortality that occurred in the late 2010s.

The estimates regarding aaCVM were pooled over a 10-year period while the social deprivation index was obtained from a single year within that study period. However, as we have detailed in a previous study, these composite county-level indices change very little over time^5^. Hence, this level of approximation would provide reliable estimates regarding county-level social deprivation over the study period. To ensure that changes in SDI would not dramatically impact mortality estimates, we obtained the SDI from two more time points within the study period and performed a sensitivity analysis on the mortality rates by SDI quartile. There is very little difference in result, as seen in Table S2.

Given the use of administrative ICD codes, we were not able to present results stratified by the stages of CKM. Therefore, we have presented the average effect that CKM syndrome has had on premature aaCVM in the US. Additionally, the AHA statement clearly highlights that the CKM syndrome is a heterogeneous condition. Due to the nature of our study design, we were not able to capture all of these nuances of CKM syndrome. Therefore, further work needs to use individual level data to evaluate the relationship between social deprivation and premature cardiovascular mortality according to different CKM phenotypes and stages.

In conclusion, age adjusted premature cardiovascular mortality rates related to CKM syndrome in the US are high with wide variation across states and counties. A large proportion of men, non-Hispanic black, and those residing in rural counties have rates much higher than the national average. The average rate in the counties in the highest quartile of social deprivation was 40% higher than the national average.

## Electronic supplementary material

Below is the link to the electronic supplementary material.

Supplementary Material 1

## Data Availability

Readers can request the data and code used in this manuscript from the corresponding authors.

## Abbreviations

CDC: Center for diseases control
IQR: Inter-quartile range
aaCVM: age adjusted cardiovascular mortality
SDI: Social deprivation index
SDoH: Social determinants of health
CKM: cardio–kidney–metabolic syndrome
