# Supplementary material for: The association between county-level premature cardiovascular mortality related to cardio-kidney-metabolic disease and the social determinants of health in the US
Source: Sci Rep. 2024 Oct 23;14:24984. doi: 10.1038/s41598-024-73974-9 (PMC11500108; doi:10.1038/s41598-024-73974-9)
Supplement: Supplementary file 1 — Supplementary Material 1 [file 41598_2024_73974_MOESM1_ESM.docx]

**The association between county-level premature cardiovascular mortality related to cardio-kidney-metabolic disease and the social determinants of health in the US.**

SUPPLEMENTARY APPENDIX

Contents

[Table S1. International Classification of Disease codes used to identify cardio-kidney-metabolic syndrome from the CDC Wonder database. 3](#_Toc171882963)

[Table S2. Sensitivity analysis for mortality rates as varied by social deprivation index year. 4](#_Toc171882964)

[Figure S1. Premature cardiovascular mortality associated with cardio-kidney-metabolic syndrome in all US states 5](#_Toc171882965)

[Figure S2. Premature cardiovascular mortality associated with cardio-kidney-metabolic syndrome in men and women residents in US states 6](#_Toc171882966)

[Figure S3. Premature cardiovascular mortality associated with cardio-kidney-metabolic syndrome in metropolitan and non-metropolitan counties in the US states 7](#_Toc171882967)

# Table S1. International Classification of Disease codes used to identify cardio-kidney-metabolic syndrome from the CDC Wonder database.

| ICD Code | Description |
| --- | --- |
| I10-I15 | Hypertensive diseases |
| I20-I25 | Ischemic heart diseases |
| I60-I69 | Cerebrovascular diseases |
| I70 | Atherosclerosis |
| I48 | Atrial fibrillation and flutter |
| I50 | Heart failure |
| N17-N19 | Renal failure |
| E11 | Non-insulin-dependent diabetes mellitus |
| E13 | Other specified diabetes mellitus |
| E65 | Localized adiposity |
| E66 | Obesity |
| E78 | Disorders of lipoprotein metabolism and other lipidaemia disorders |

We present the international classification of disease 10^th^ version codes that we used in the ‘multiple causes of death’ field to identify cardio-kidney-metabolic syndrome in the Center for Diseases Control Wide-ranging ONline Data for Epidemiologic Research (WONDER) data portal.

# Table S2. Sensitivity analysis for mortality rates as varied by social deprivation index year.

|  | SDI Quartile 1 | SDI Quartile 2 | Q2 vs Q1 p-value | SDI Quartile 3 | Q3 vs Q2 p-value | SDI Quartile 4 | Q4 vs Q3 p-value |
| --- | --- | --- | --- | --- | --- | --- | --- |
| aaCVM per Quartile using 2012 SDI | 44.7 (36.2 - 54.9) | 60.8 (48.8 - 76.9) | < 0.01 | 77.3 (61.4 - 93.9) | < 0.01 | 88.7 (70.0 - 110.4) | < 0.01 |
| aaCVM per Quartile using 2016 SDI | \| 44.7 (36.1 - 54.6) \| \| --- \| | 59.2 (47.5 - 73.8) | < 0.01 | 75.8 (60.2 - 92.6) | < 0.01 | 91.1 (71.5 - 111.9) | < 0.01 |
| aaCVM per Quartile using 2018 SDI | 44.1 (35.8 - 53.4) | 59.0 (47.5 - 73.4) | < 0.01 | 76.5 (60.2 - 93.9) | < 0.01 | 90.7 (71.3 - 111.0) | < 0.01 |

This table presents the age-adjusted premature cardiovascular mortality rates (aaCVM)(per 100,000 residents; 2010 – 2019) associated with cardio-kidney-metabolic syndrome according to the social deprivation index in the US. We fitted these models using the social deprivation index (SDI) from years 2016 & 2018 and then compared these results with our primary analysis using the 2012 SDI. Pairwise comparison were fitted by the Wilcoxon test and Bonferroni correction.

# Figure S1. Premature cardiovascular mortality associated with cardio-kidney-metabolic syndrome in all US states


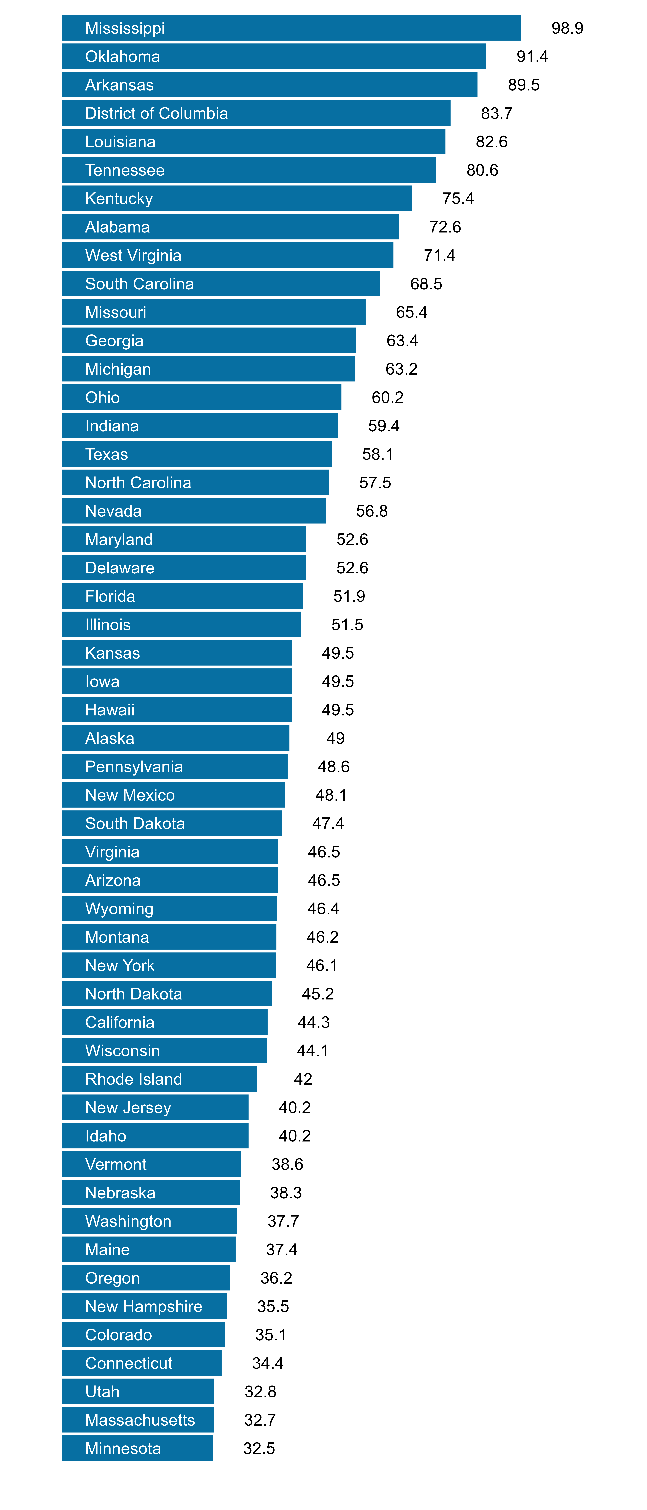


Rate per 100,000 residents

State

This figure depicts the premature cardiovascular mortality related to cardio-kidney-metabolic syndrome per 100,000 residents between 2010 and 2019 in all the US states. The overall median premature cardiovascular mortality rate related to cardio-kidney-metabolic syndrome between 2010 and 2019 in the US was 60.6/100,000 residents.

# Figure S2. Premature cardiovascular mortality associated with cardio-kidney-metabolic syndrome in men and women residents in US states

State

| 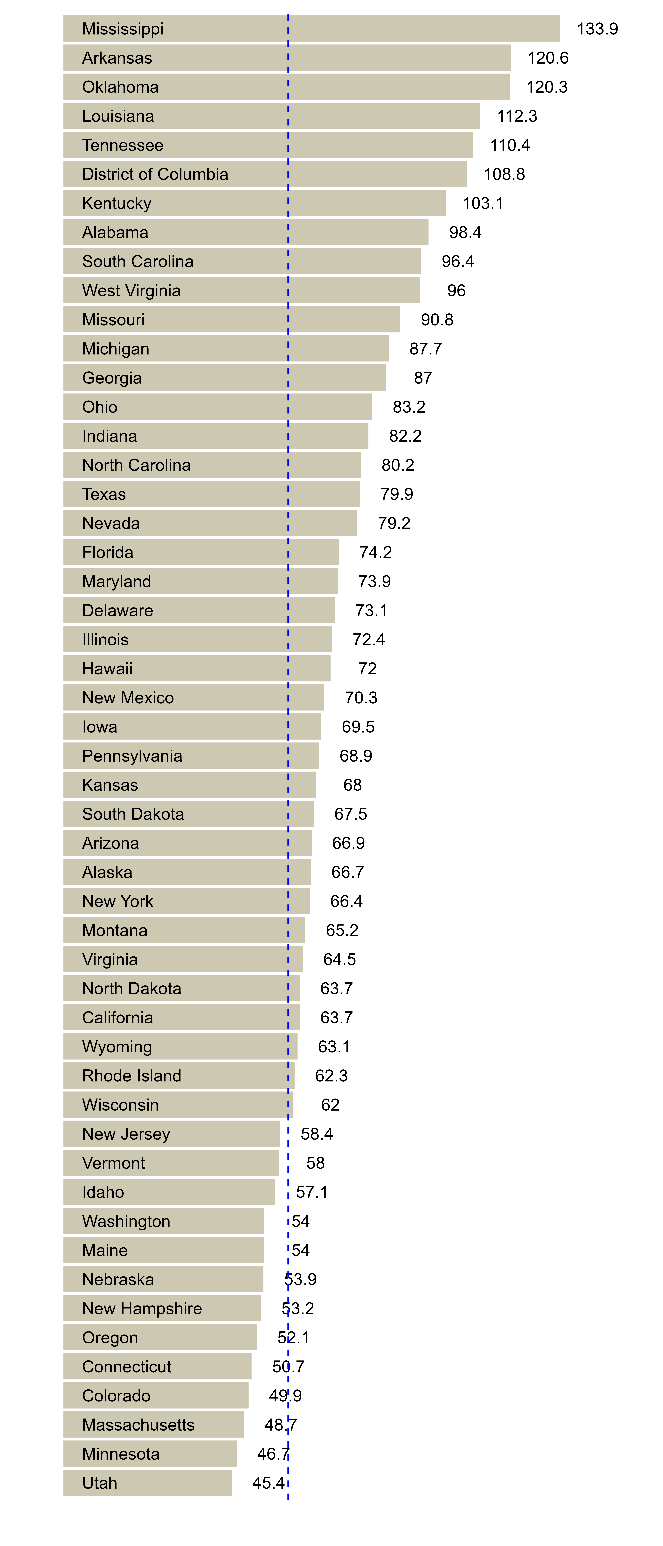  Rate per 100,000 residents | 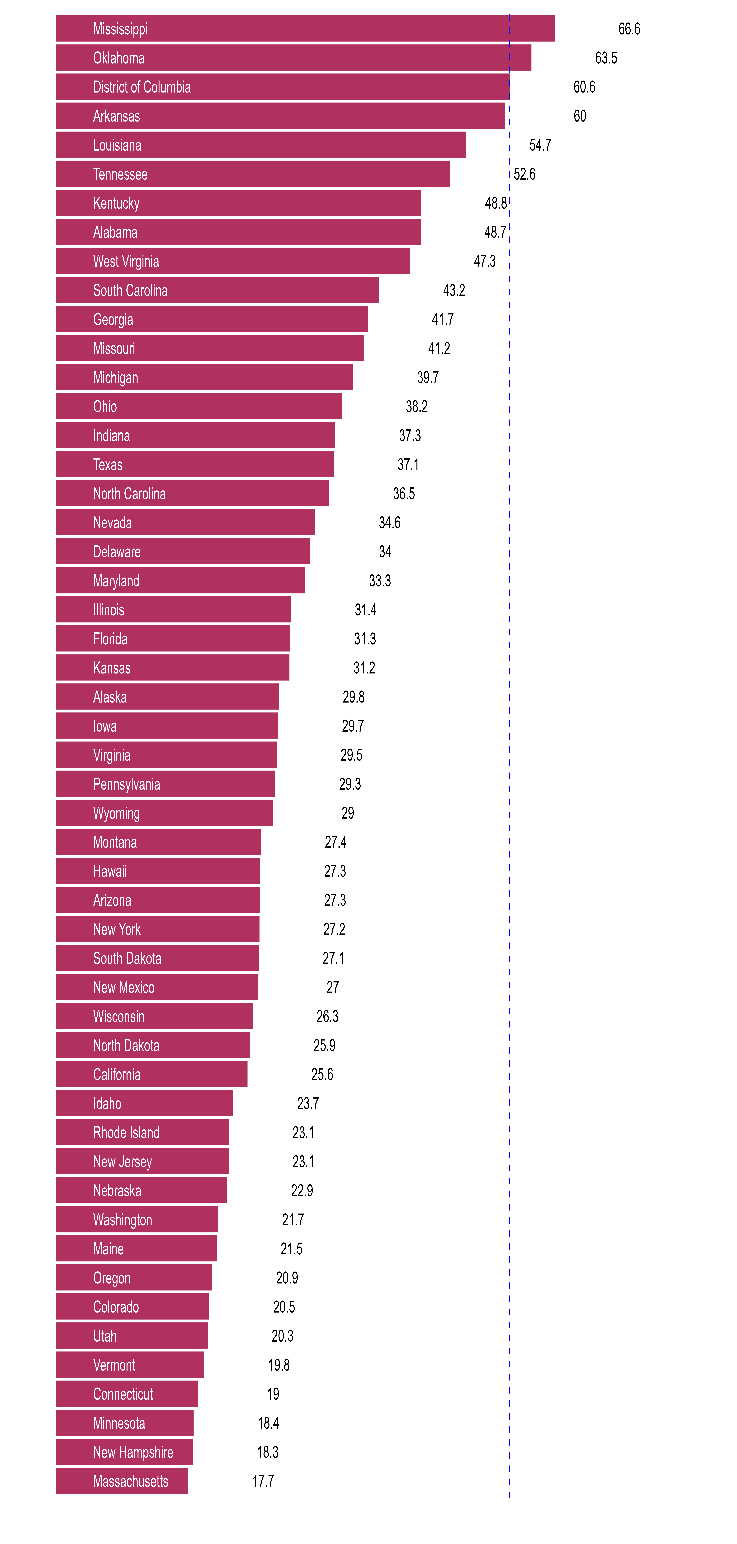  State  Rate per 100,000 residents |
| --- | --- |
| A) This figure depicts the premature cardiovascular mortality rates (per 100,000 residents) related to cardio-kidney-metabolic syndrome (2010-2019) for men in US states. The dashed line presents the median overall rate (60.6/100,000 residents) for cardio-kidney-metabolic syndrome related premature cardiovascular mortality in the US | B) This figure depicts the premature cardiovascular mortality rates (per 100,000 residents) related to cardio-kidney-metabolic syndrome (2010-2019) for women in US states. The dashed line presents the median overall rate (60.6/100,000 residents) for cardio-kidney-metabolic syndrome related premature cardiovascular mortality in the US |

# Figure S3. Premature cardiovascular mortality associated with cardio-kidney-metabolic syndrome in metropolitan and non-metropolitan counties in the US states

| 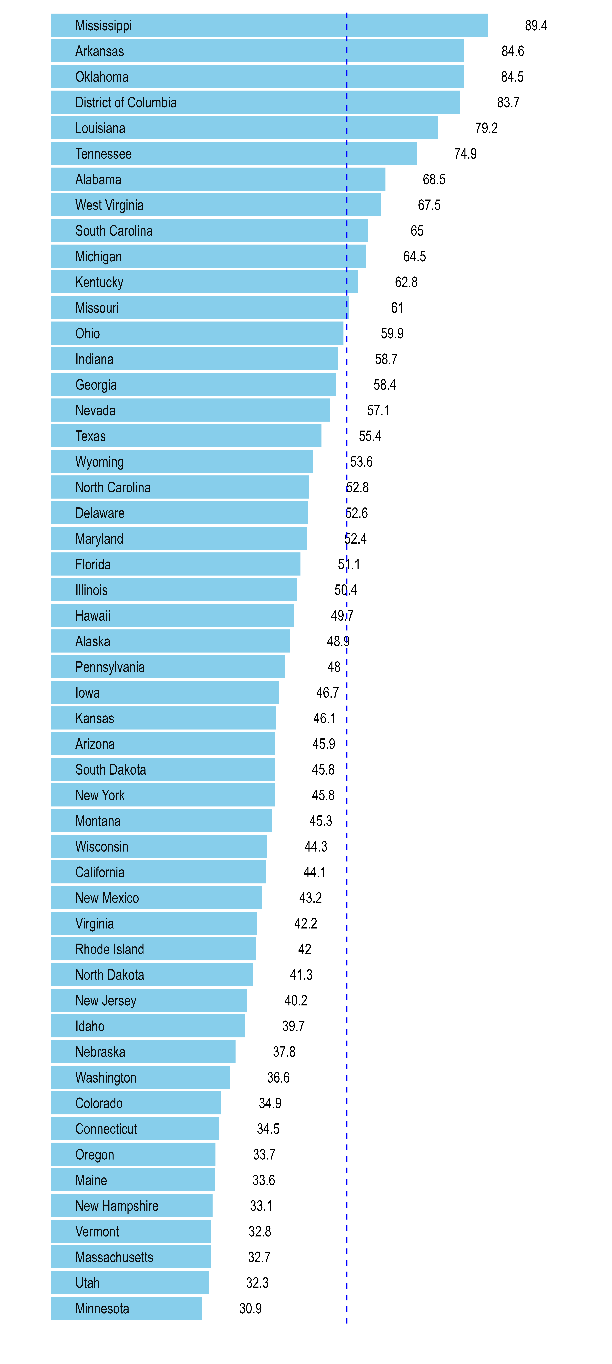  Rate per 100,000 residents  State | 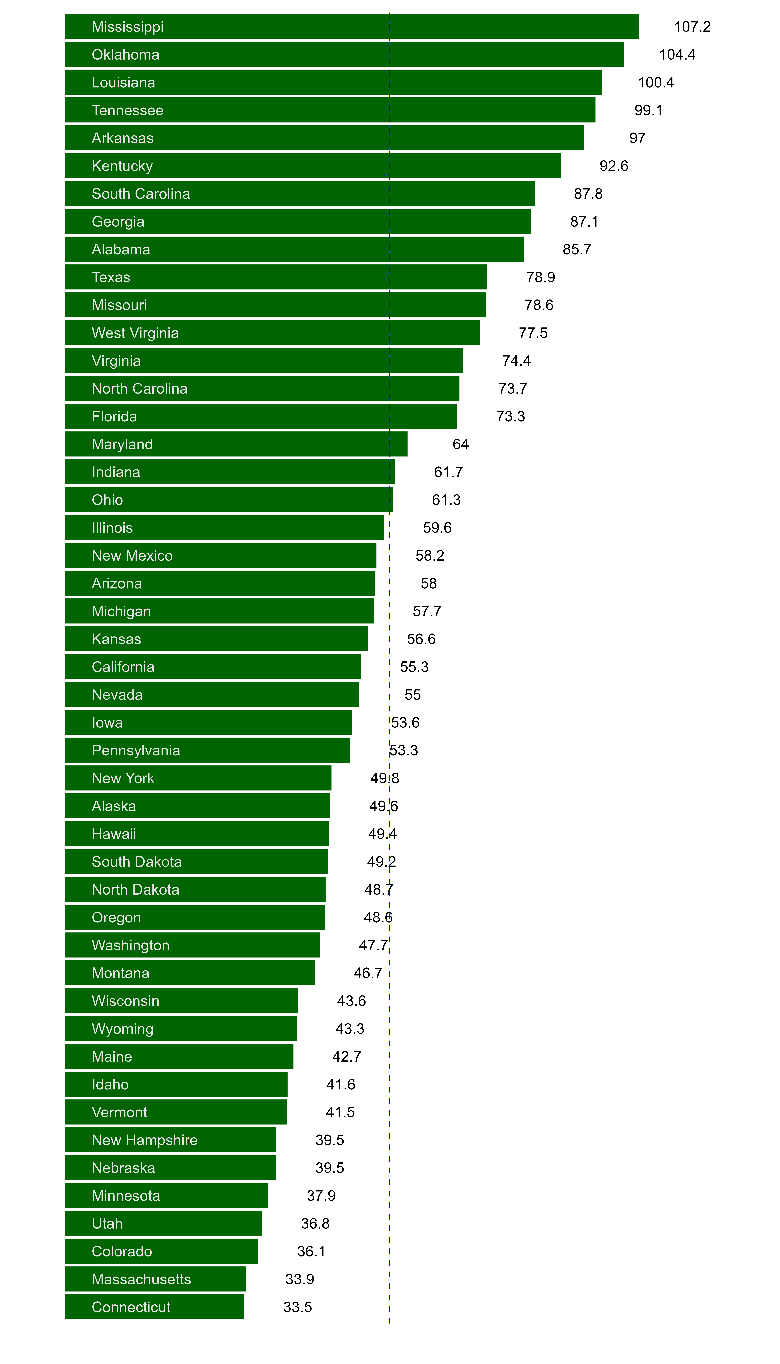  State  Rate per 100,000 residents |
| --- | --- |
| A) This figure depicts the premature cardiovascular mortality rates (per 100,000 residents) related to cardio-kidney-metabolic syndrome (2010-2019) for metropolitan counties in US states. The dashed line presents the median overall rate (60.6/100,000 residents) for cardio-kidney-metabolic syndrome related premature cardiovascular mortality in the US | B) This figure depicts the premature cardiovascular mortality rates (per 100,000 residents) related to cardio-kidney-metabolic syndrome (2010-2019) for metropolitan counties in US states. The dashed line presents the median overall rate (60.6/100,000 residents) for cardio-kidney-metabolic syndrome related premature cardiovascular mortality in the US |
